# Supplementary material for: High Intrinsic Oncogenic Potential in the Myc-Box-Deficient Hydra Myc3 Protein
Source: Cells. 2023 Apr 26;12(9):1265. doi: 10.3390/cells12091265 (PMC10177328; doi:10.3390/cells12091265)
Supplement: Supplementary file 1 [file cells-12-01265-s001.zip › cells-2296436-supplementary.pdf]

Supplementary Material

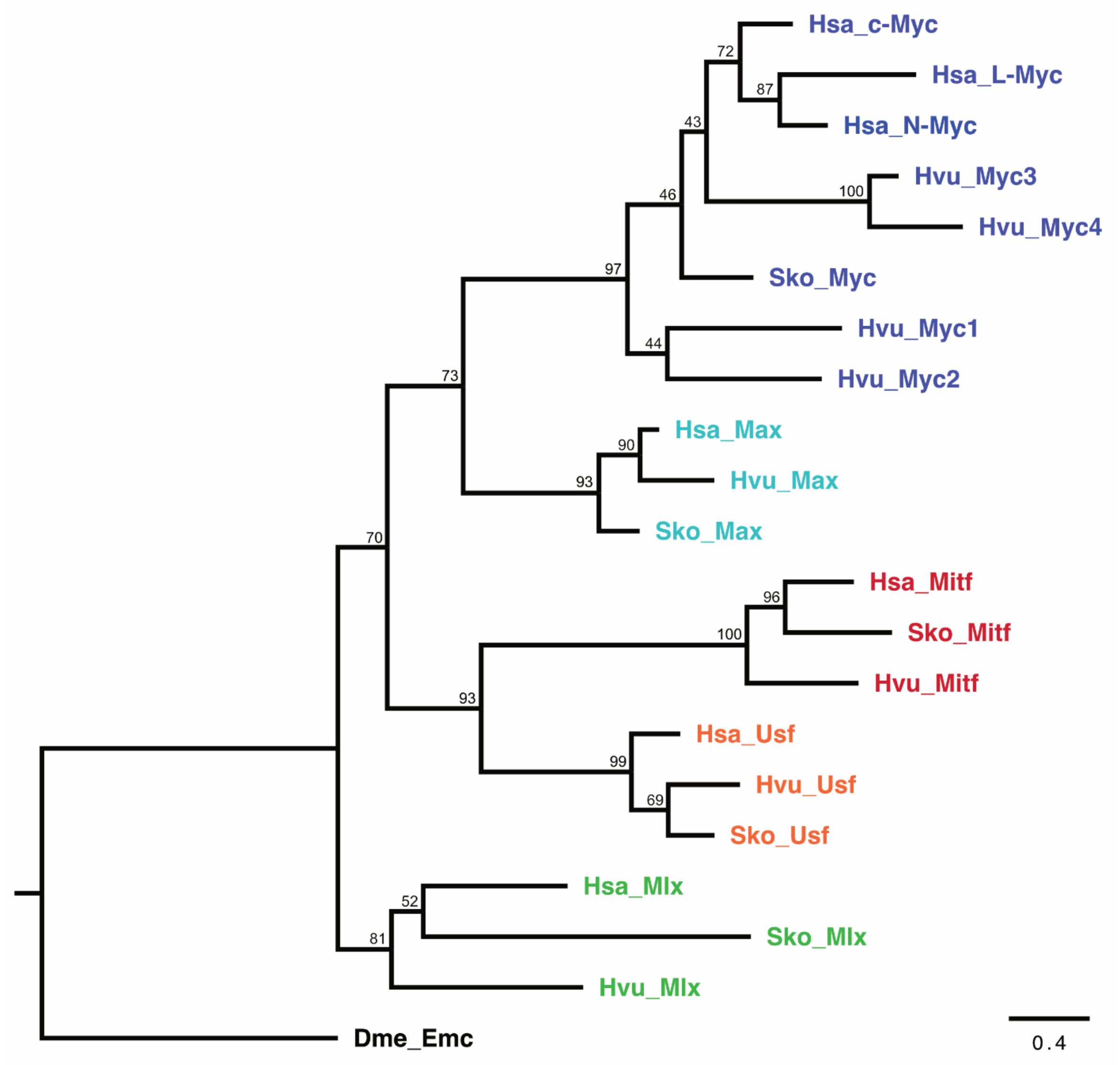

Figure S1. Phylogenetic analysis of selected transcription factor subfamilies using their bHLH-LZ domain amino acid sequences. The phylogenetic tree resolves the subfamily clusters of transcription factors of the Myc-Max-Mlx network and of related Mitf (microphthalmia-associated transcription factor) and Usf (upstream stimulary factor) proteins, and places the C-terminal domains of *Hydra* Myc3 and Myc4 within the Myc subfamily cluster. Color code: Myc: blue; Max: cyan; Mitf: red; Usf: orange; Mlx: green. In order to compare with *Hydra* (Hvu, *Hydra vulgaris*) homologs, proteins were selected from human (Hsa, *Homo sapiens*) and *Saccoglossus* (Sko, *Saccoglossus kowalevskii*), because vertebrates and members of hemichordate invertebrates are regarded to represent only moderately derived taxa. The different MYC isoforms in human (c-Myc, L-Myc, N-Myc) and in *Hydra* (Myc1-4) are included in the analysis. Accession numbers: Hsa_c-myc, XP_001129632; Hsa_L-Myc, NP_001028254; Hsa_N-Myc, XP_006711949; Sko_Myc, NP_001158444; Hvu_Myc1, XP_002163473; Hvu_Myc2, XP_002164060; Hvu_Myc3, XP_002170328; Hvu_Myc4, XP_012556510; Hsa_Max, NP_001394032; Sko_Max, XP_002737628; Hvu_Max, XP_002167227; Hsa_Mitf, NP_937821; Sko_Mitf, NP_001161587; Hvu_Mitf, XP_047126457; Hsa_Usf, NP_001263302; Sko_Usf, XP_006820038; Hvu_Usf, XP_012566918; Hsa_Mlx, NP_937848; Sko_Mlx, XP_006815558; Hvu_Mlx, XP_047141571. Amino acid sequences of the C-terminal bHLH-LZ domains were subjected to MAFFT alignment [1], and a maximum likehood tree was inferred using the phylogenetic tool W-IQ-TREE [2]. Branch support values were obtained with 1,000 Bootstrap alignments (correlation coefficient > 0.99; maximum iterations: 1,000; perturbation strength: 0.8). The output tree, rooted using the *Drosophila melanogaster* bHLH protein extra macrochaetae (Dme_Emc, accession number NP_523876), was visualized and annotated using the program FigTree v1.4.4 (http://tree.bio.ed.ac.uk/). The scale bar represents the expected number of amino acid substitutions per site.

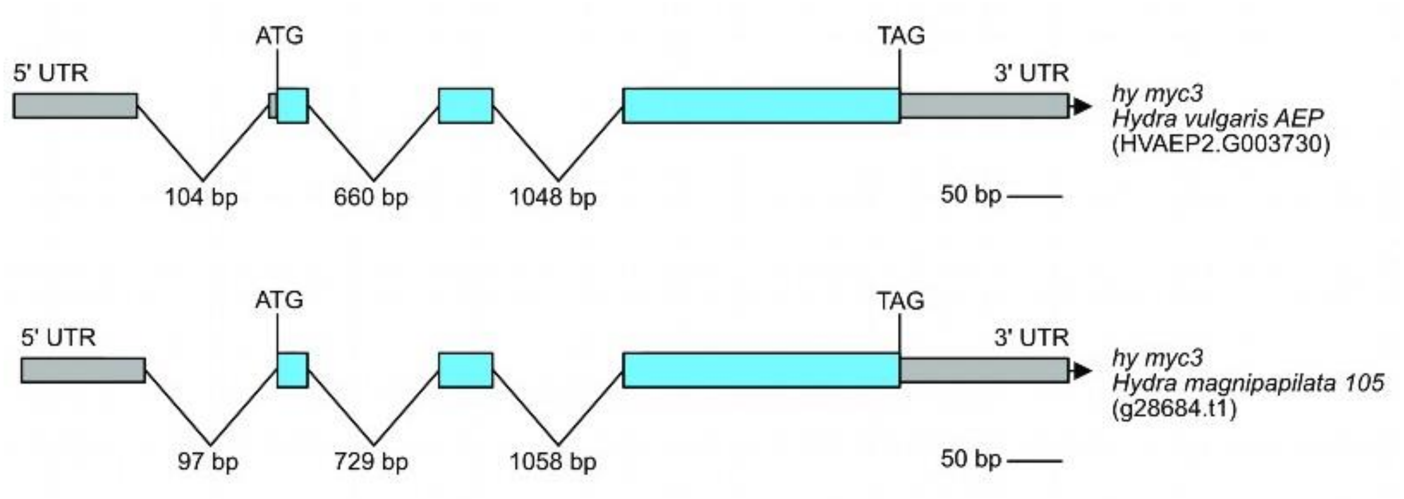

Figure S2. Topographies of the *myc3* genomic loci in the strains *Hydra vulgaris* AEP and *Hydra magnipapillata 105*. Each locus is positioned on chromosome 2 (https://research.nhgri.nih.gov/HydraAEP/sequenceserver, accession no. HVAEP2.T003730). Exons are represented in grey, and the coding regions in blue with indicated start (ATG) and stop codons (TAG).

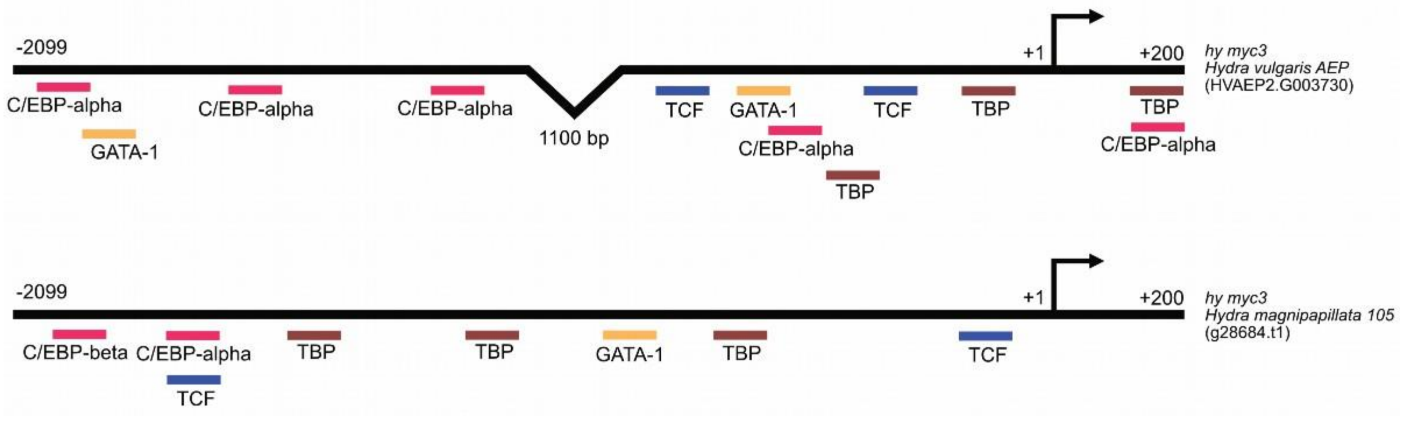

Figure S3. Promoter maps of *Hydra myc3* in the strains *Hydra vulgaris* and *Hydra magnipapillata* 105. Schematic representation of transcription factor binding sites. The bars represent the promoter regions and the arrows depict the transcription start sites (+1). The positions of selected transcription factor binding sites encoded in both strains as identified by the computer program AliBaba2 (gene-regulation.com) are presented below the bar (C/EBP-alpha, CCAAT enhancer-binding protein-alpha; GATA-1, GATA binding factor 1; TBP, TATA box-binding protein; TCF, T-cell-specific transcription factor).

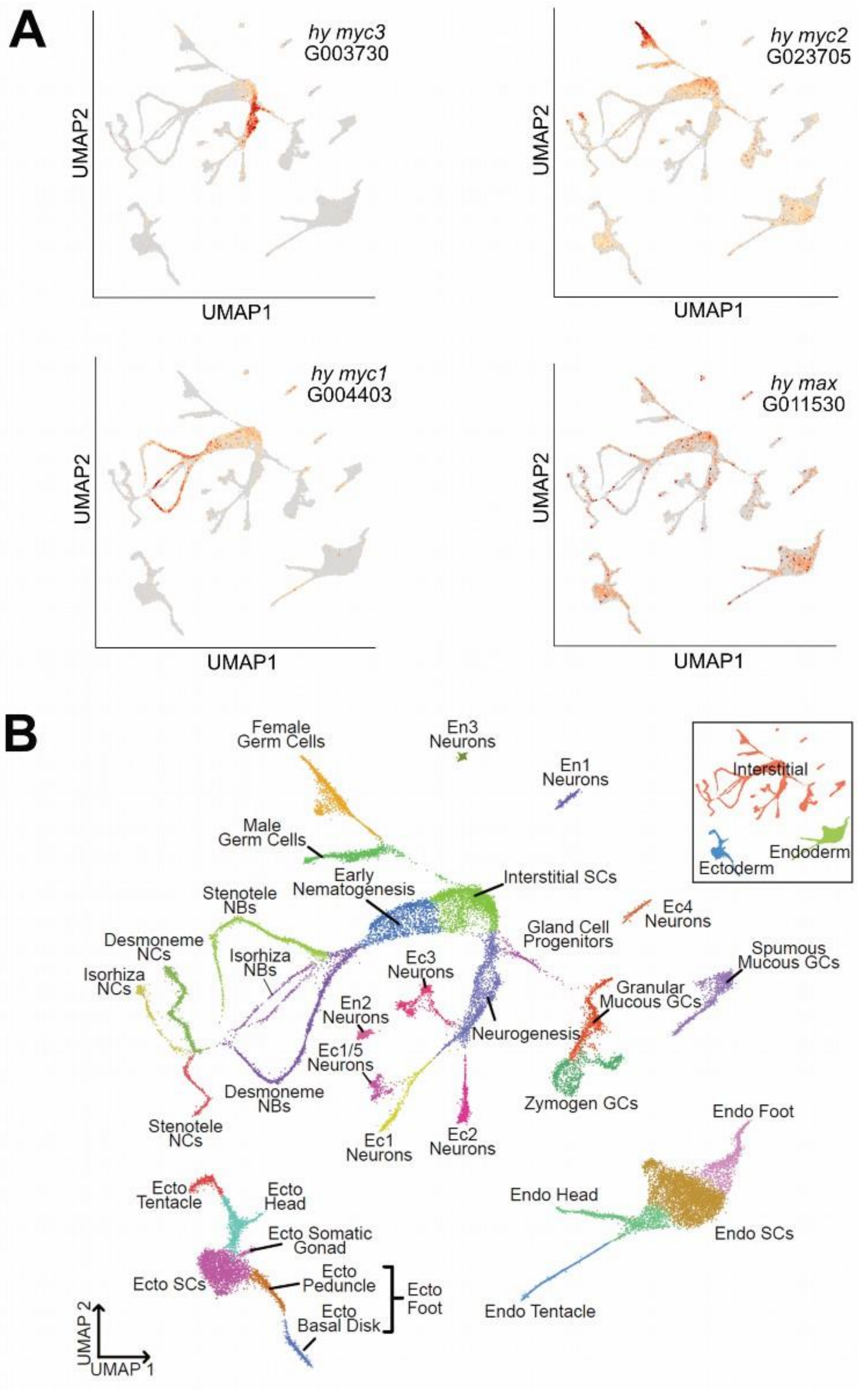

Figure S4. Single cell expression of *Hydra* (hy) *myc1*, *myc2*, *myc3* and of *Hydra max*. (**A**) UMAP representation of *myc3*, *myc2*, *myc1* and *max* expression in the whole animal single-cell atlas [3, 4]. *myc3* shows specific expression in stem cell progenitors committed to nerve and gland cell differentiation. *myc2* is widely expressed in interstitial stem cells, early nematoblasts, gametes, gland cells and both ectodermal and endodermal cells. *myc1* expression is restricted to interstitial stem cells and nematoblasts. *max* is expressed in all cells thereby overlapping the expression of all three *myc* variants. (**B**) Single cell atlas with annotated cell clusters [3].

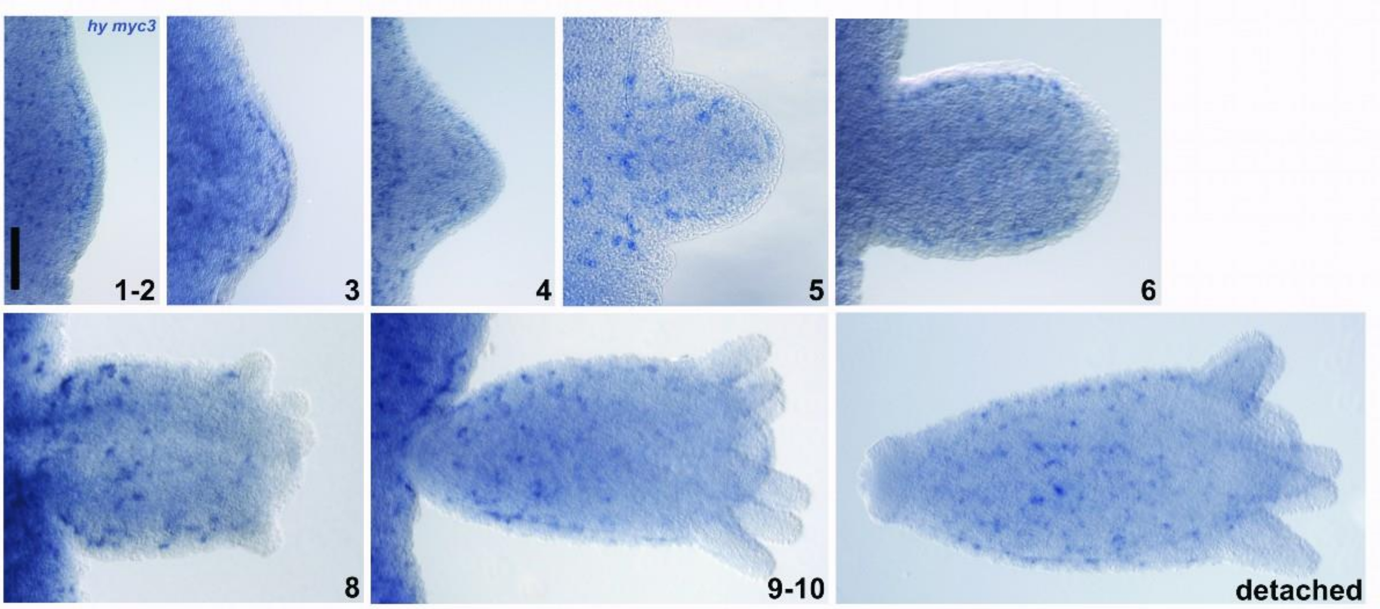

Figure S5. Expression of *Hydra myc3* during asexual bud formation visualized by *in situ* hybridization. In early bud stages (one to three), *myc3*-expressing precursor cells accumulate at the site of tissue evagination as described previously [5]. Beginning with stage four, these precursor cells disappear from the oral tip and from the newly forming mouth opening. In late stages (eight to ten) and freshly detached buds, *myc3*-expressing precursor cells are located in the gastric region, but not in the terminally differentiated head (hypostome plus tentacles) and foot (basal disc) tissues. Bud stages correspond to those defined by Otto & Campbell [6]. Scale bar: 200 μm.

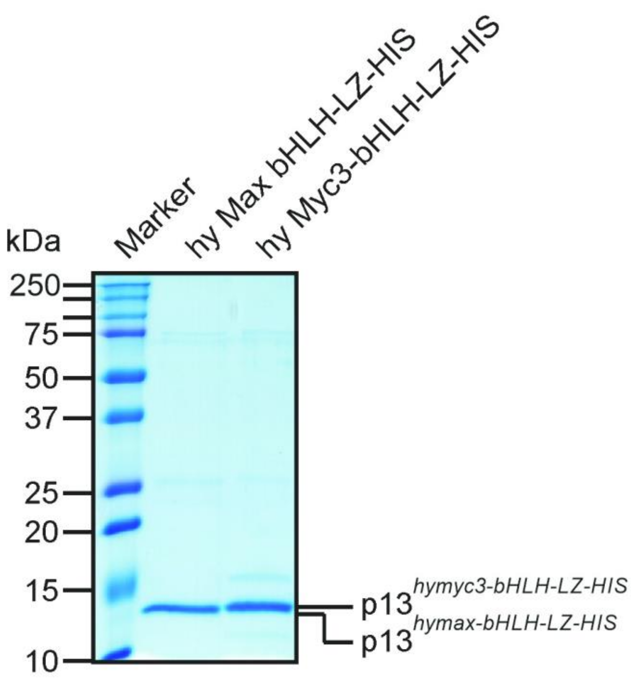

Figure S6. Recombinant protein analysis. SDS-PAGE (14% gradient, wt/vol) of 1-μg aliquots of affinity-purified recombinant *Hydra* Max bHLH-LZ-HIS and Myc3 bHLH-LZ-HIS p13 proteins. The gel was stained with Coomassie brilliant blue.

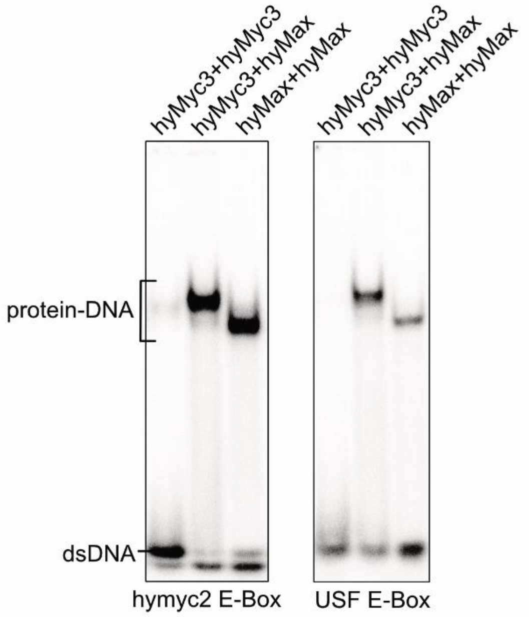

Figure S7. Interactions of Myc3 or Max homodimers, and of Myc3/Max heterodimers with canonical Myc binding sites. Electrophoretic mobility shift assays (EMSA) using recombinant polypeptides (p13) encompassing the *Hydra* Myc3 or Max bHLH-LZ regions (2 μM), and each 0.3-ng (25,000 cpm) aliquots of a ^{32}P-labeled double-stranded 18-mer oligodeoxynucleotide containing either the canonical Myc/Max- binding motif 5′-CACGTG-3′ (E-box) in the context of the *Hydra myc2* promoter [7] (left panel), or in the context of the upstream stimulatory factor binding site (E-box USF) [8] (right panel).

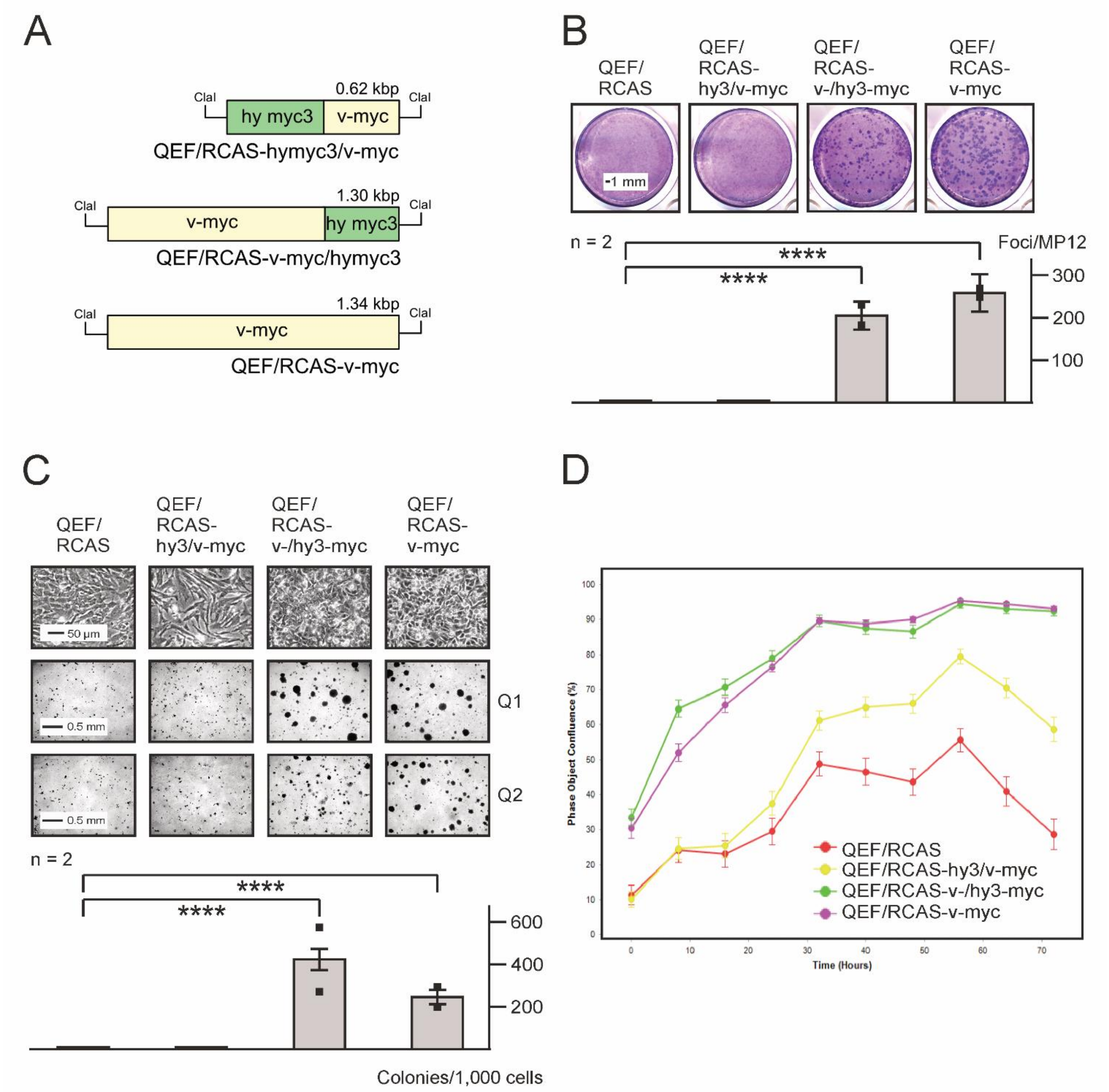

Figure S8. Transforming activities of *Hydra* (hy) and viral (v) Myc hybrid proteins. (**A**) Schematic depiction of the v-*myc* (yellow) and of hy3/v-*myc* and v-*myc*/hy3 hybrids' coding regions (*Hydra myc3* sequences are shown in green; bHLH-Zip, basic/helix-loop-helix/leucine zipper region). The coding regions were inserted into the unique *Cla*I site of the replication-competent retroviral pRCAS vector used for DNA transfection into quail embryo fibroblasts (QEF). (**B**) Cell transformation assays of QEF transfected with the RCAS constructs depicted under (A). Each 1 µg of plasmid DNA were transfected into QEF grown onto MP12 wells and then kept under agar overlay for 2 weeks followed by staining with eosin methylene blue (upper panel). Foci were counted on MP12 dishes (n = 2). Vertical bars show standard deviations (SD) from triplicates (lower panel). Statistical significance was assessed by using a paired Student's t-test (****$P < 0.0001$). (**C**) For mass cultures, each 4 µg of plasmid DNA were transfected into QEF grown on 60-mm dishes. Cells were passaged several times and phase-contrast micrographs were taken to visualize cell morphologies (upper panel). Equal numbers of these cells (1.25×10^4) were seeded into soft agar on MP24 wells and incubated for 2 weeks. Numbers of colonies per 1,000 cells seeded are shown next to the bright-field micrographs. Vertical bars show standard deviations (SD) from triplicates (lower panel). Statistical significance was assessed by using a paired Student's t-test (****$P < 0.0001$). (**D**) Proliferation of cells described under (C). Each 1.25×10^5 cells were seeded in triplicate onto 24-well cell culture plates and cell densities measured every 8 h over a 3-day time period using an IncuCyte live-cell analysis system.

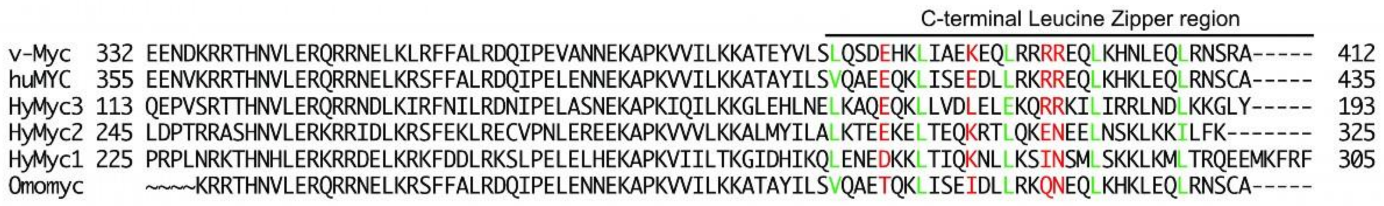

Figure S9. Protein alignment of Myc bHLH-LZ regions. The C-terminal domains from v-Myc and human c-Myc were compared to the corresponding regions from the *Hydra* Myc variants, and the Myc competitor peptide Omomyc. *Hydra* Myc3 has more similarities to v-Myc/c-Myc in comparison to *Hydra* Myc2 and Myc1. Residues in red play a role in the interaction between Myc and Max in the leucine zipper region, according to interaction profile analyses (cf. Figure 5) and to previous data [9]. Leucine zipper-relevant residues are highlighted in green.

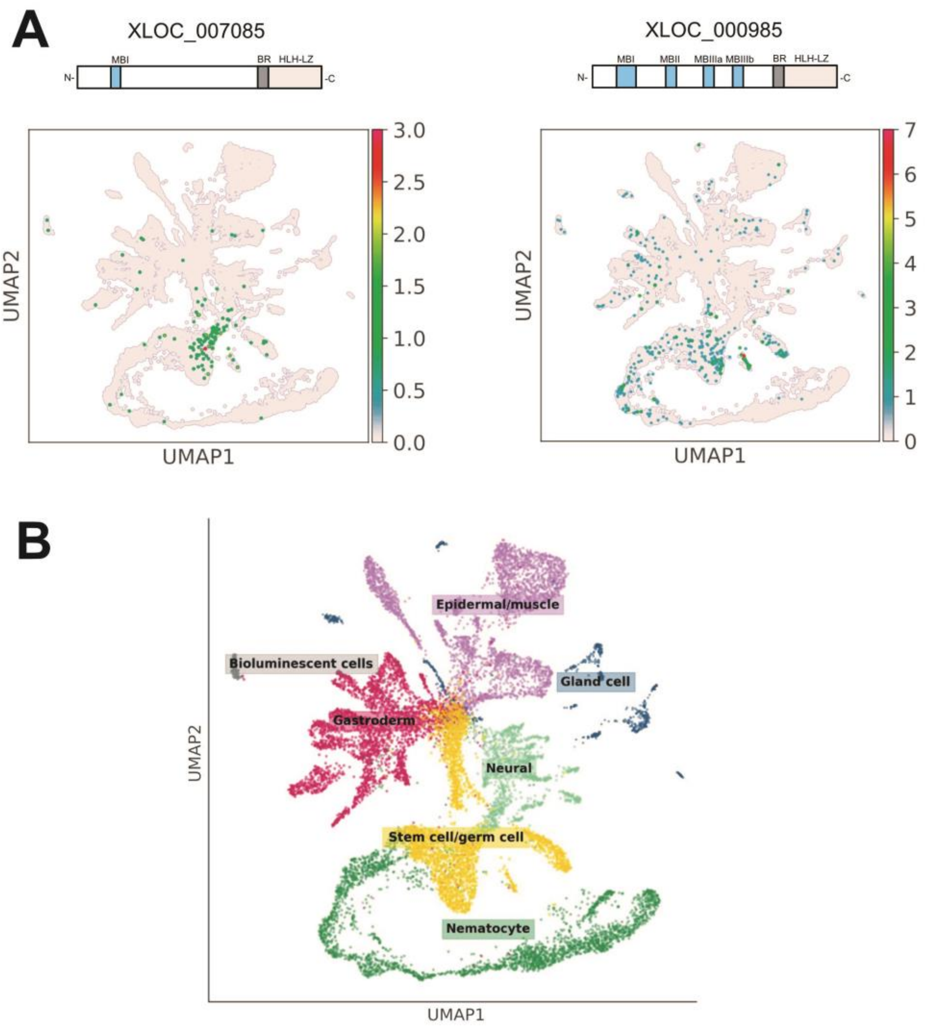

Figure S10. Single cell expression of two *Clytia hemisphaerica myc* paralogs. (**A**) UMAP representation of XLOC_007085 and XLOC_000985. The predicted XLOC_007085 protein exhibits a Myc-specific C-terminal domain, but its N-terminal part is derived and contains only Myc box I. The corresponding gene is primarily expressed during nerve cell differentiation. The predicted XLOC_000985 protein is similar to *Hydra* Myc2 in its domain organization, and the encoding gene shows a broad expression pattern in interstitial stem cells, early stages of nematocyte differentiation, gametes, and ectodermal and endodermal epithelial cells. (**B**) Single cell atlas with annotated cell type clusters [10].

References

1. Kuraku S, Zmasek CM, Nishimura O, Katoh K. aLeaves facilitates on-demand exploration of metazoan gene family trees on MAFFT sequence alignment server with enhanced interactivity. Nucleic Acids Res. 2013;41(Web Server issue):W22-8 DOI: 10.1093/nar/gkt389.
2. Trifinopoulos J, Nguyen LT, von Haeseler A, Minh BQ. W-IQ-TREE: a fast online phylogenetic tool for maximum likelihood analysis. Nucleic Acids Res. 2016;44(W1):W232-5 DOI: 10.1093/nar/gkw256.
3. Cazet JF, Siebert S, Little HM, Bertemes P, Primack AS, Ladurner P, et al. A chromosome-scale epigenetic map of the Hydra genome reveals conserved regulators of cell state. Genome Res. 2023;33(2):283-98 DOI: 10.1101/gr.277040.122.
4. Siebert S, Farrell JA, Cazet JF, Abeykoon Y, Primack AS, Schnitzler CE, et al. Stem cell differentiation trajectories in Hydra resolved at single-cell resolution. Science. 2019;365(6451) DOI: 10.1126/science.aav9314.
5. Berking S. Commitment of stem cells to nerve cells and migration of nerve cells precursors in preparatory bud development in Hydra. J Embryol Exp Morphol. 1980;60:373-87 DOI: 10.1242/dev.60.1.373.
6. Otto JJ, Campbell RD. Budding in Hydra attenuata: bud stages and fate map. J Exp Zool. 1977;200(3):417-28 DOI: 10.1002/jez.1402000311.
7. Hartl M, Glasauer S, Gufler S, Raffeiner A, Puglisi K, Breuker K, et al. Differential regulation of myc homologs by Wnt/beta-Catenin signaling in the early metazoan Hydra. FEBS J. 2019;286(12):2295-310 DOI: 10.1111/febs.14812.
8. Hartl M, Mitterstiller AM, Valovka T, Breuker K, Hobmayer B, Bister K. Stem cell-specific activation of an ancestral myc protooncogene with conserved basic functions in the early metazoan Hydra. Proc Natl Acad Sci U S A. 2010;107(9):4051-6 DOI: 10.1073/pnas.0911060107.
9. Soucek L, Whitfield J, Martins CP, Finch AJ, Murphy DJ, Sodir NM, et al. Modelling Myc inhibition as a cancer therapy. Nature. 2008;455(7213):679-83 DOI: 10.1038/nature07260.
10. Chari T, Weissbourd B, Gehring J, Ferraioli A, Leclere L, Herl M, et al. Whole-animal multiplexed single-cell RNA-seq reveals transcriptional shifts across Clytia medusa cell types. Sci Adv. 2021;7(48):eabh1683 DOI: 10.1126/sciadv.abh1683.
